# Detecting cocaine use? The autobiographical implicit association test (aIAT) produces false positives in a real-world setting

**DOI:** 10.1186/1747-597X-8-22

**Published:** 2013-06-14

**Authors:** Elisabeth Julie Vargo, Andrea Petróczi

**Affiliations:** 1 School of Life Sciences, Kingston University, Kingston upon Thames Surrey KT1 2EE, UK

**Keywords:** Lie-detector, Cocaine use, Implicit Association Test, Craving, Memory, Mixed methodology, Touch-screen technology

## Abstract

**Background:**

The autobiographical Implicit Association Test (aIAT) is a novel application of the implicit association concept for detecting life events. It has been used to reveal concealed knowledge in clinical and forensic settings, including detecting drug use. In this study, we aimed to explore the functionality of the aIAT to identify drug use in real-world settings.

**Methods:**

The study used mixed methodology with known groups of drug users and nonusers. Recreational cocaine users (n = 23) and non-users (n = 23) were recruited through ethnographic methodology and assessed using a bespoke brief aIAT for cocaine use. An identical aIAT test for heroin detection was also administered to a sub-sample of 10 cocaine users and 13 nonusers. The accuracy of the cocaine aIAT was measured through ROC analysis. Paradoxical aIAT results were explored by integrating craving, consumption measures and life-story interviews into the analysis.

**Results:**

Whilst the two brief aIATs showed good concurrent validity for cocaine users by accurately detecting drug using status for 18 of the 23 users (78.3%), the test falsely reported 61% cocaine users in the non-user comparison group. The average D-scores were 0.257±0.246 for the cocaine users and 0.134±0.367 for the non-users, showing no discriminatory power (t(44) = 1.339, p = 0.187; AUC = 0.605, p = 0.223). Results were independent from craving and recent cocaine use. The comparison group’s cocaine and heroin aIAT scores correlated significantly (r(13) = 0.776, p = 0.002) whilst an accurate absence of such relationship was evidenced in the cocaine using sample (r(10) = 0.061, p = 0.866). Triangulation with life-story interviews suggests that in the absence of an autobiographical event, this test may measure an alternative cognitive construct linked to the Self-concept.

**Conclusion:**

The aIAT is a variant of an attitude measure and can be better rationalized if propositional thinking is implied to explain outcomes. The Relational Frame and Social Knowledge Structure theories can perhaps provide a more plausible theoretical background. Further work is required to clarify which factors underlie this testing technique’s functioning. Reappraisal is advised before further forensic use of the instrument to ensure that general associations not related to autobiographical memory do not confound results.

## Background

After more than two decades of research into implicit social cognition, Nosek and Riskind persuasively argue that social policies aiming to influence, change or form people’s behaviour could be ineffective if they do not take people’s inner thoughts (their implicit cognition) into account [1]. Consciously held and explicitly reported information can only provide a narrow access into people’s thought processes. In reality, behaviours do not result exclusively from conscious intents; nor are people always capable of giving an accurate account of the factors that led to their behavioural choices. Stemming from social cognitive psychology, practical applications of implicit cognition research have proliferated over the recent years and gained strong footholds in other disciplines such as law, substance abuse, healthcare, criminology, forensics and marketing [2-4].

The Implicit Association Test (IAT) [5] is commonly accepted as a reliable implicit response time measure [6]. Its applicability has contributed to an exponential increase of research attempting to assess various implicit constructs [2-4]. Applications of response-time measure tests as lie detectors [7,8] imply the use of an indirect computerized sorting task for the recollection of autobiographical memories with the view of detecting concealed life events. One of the two existing IAT-based ‘lie detector’ tests is the autobiographical Implicit Association Test (aIAT) [8], which has been applied to clinical and forensic cases outside academia and has been the subject of numerous investigations since its conception. The notable increase in clinical, forensic and experimental applications of such tests [9-12] warrants investigation into the performance of these outside laboratory experiments to establish validity, especially when these instruments are used in real-world settings [13].

The aIAT compares response times in associating the label ‘*True*’ to mutually exclusive categories of autobiographical events. A person’s specific autobiographical event can be identified by comparing their speed in matching generically true sentences (e.g. *I am in front of the computer*) and potentially true statements (e.g. *I snort cocaine*) with labels ‘*True*’ or ‘*False*’ [8]. Autobiographical events are operationally defined as “an individual’s ability to remember events he or she has experienced directly” p772 [8]. Although this method is declared capable of detecting concealed knowledge with high (> 90%) diagnostic accuracy [14,15], Sartori and colleagues’ [8] studies reported incongruent results which led us to re-examine the experimental methodologies and further explore the functioning of this technique. In particular, the application of the aIAT to detect cocaine and heroin use [8] showed significantly higher accuracy for the heroin abusing group compared to the cocaine abusing one. It was suggested that differences in cognition [16] caused by cocaine abuse, could have led to such probabilistically bizarre results (i.e. lower D-scores for the cocaine abusing group). Independently, research has also demonstrated that associations between representations of the Self and heroin use are stronger in individuals who are heavily dependent [17].

However, these results could equally suggest that the experimental methodology used by the test developers is limiting our comprehension of the instrument. Notably, original authors never examined the aIAT technique through the comparison of results deriving from comparison samples naïve to the target behaviour [8,14,15]. As the fundamental question for the aIAT’s validity is the ‘autobiographical event’ variable, the main issue remains the exact cognitive nature of an autobiographical event targeted by the aIAT. Considering that the outcome variable is defined as an autobiographical memory of a life event experienced directly by the individual, the test would then work on the principle that preferences in matching imply unconscious involvement, and that memory can be discriminated from other affective processes. To date, the exact entity of IAT outcomes remains unclear [6], along with various unexplored issues regarding the reliability of implicit measures [18].

### Aims

In the present study, the exploration of participant characteristics and socio-psychological variables is seen as an implementation to the analysis of indirect cognitive measures. This is in contrast with the traditional approaches used for these types of explorations, which typically maintain consistently structured research designs and limit interpretation to quantitative data [6]. This intent was pursued with the aid of mixed methods, which contributed to a consistent increase in data available for interpretation, as well as to the expansion of the range obtainable from a valuable but constrained participant group.

The primary aim of this study was to test the aIAT in a real world setting, focusing on its functionality for identifying drug using populations. Considering that the sorting task is directly based upon Implicit Association Testing [5], and confusion remains regarding the construct which is assessed by this technique as well as for other implicit measures [6], this project set out to re-examine the instrument through the limitations identified by the test developers themselves. In particular, we set out to investigate whether the use of negative statements elicited ‘salience asymmetrics’ interfering with response times [19] and if consumption characteristics had any effect on performance [8]. Bearing in mind the impossibility of assessing biochemically if an individual has used an illicit substance during their whole lifetimes, the creation of a co-participative relationship with the participant sample was fundamental in the collection of reliable data [20].

## Methods

The study utilized a mixed method design featuring two sequential experimental studies and interviews in ecological settings. Qualitative data from self-reports and life story interviews were used to aid the interpretation of quantitative results obtained from the aIATs. The ethnographic recruitment of known groups of cocaine users and non-users provided access to informants who actively participated to the research process, filling the gap between the need of objective indicators relative to drug using conducts, and the exploration of a hidden population [21].

A challenge for this validation study, in fact, was establishing the absence of a substance in a person’s whole lifetime. This cannot be accomplished with the aid of biochemical testing. Although cocaine and its metabolites are readily detectable in bio-samples, evidence for consumption is only available for a limited period of time [22,23]. Cocaine metabolites can be present up to 1 year in head hair [24], assuming that the required hair length (approximately 12–14 cms) is available. Theoretical sampling [25] is more considerate of ecological factors and lets the researcher exclude participants whose self-reports are considered less reliable. Therefore, the use of mixed methods and a person-centred approach permitted to obviate this limitation and explore the validity of the aIAT in real-world settings.

### Research process

The research process comprised of several incremental steps: firstly, a brief version of the aIAT was modelled following the method originally set by Sartori et al. [8] and Sriram & Greenwald [26], taking other indications from the pertinent literature into consideration. Considering the significant differences to Sartori’s work where sample and setting follow the criteria of classical, *in situ* test administration [8,15,27], the present study attempted to create a qualitative background for the interpretation of indirect quantitative measures [28].

The bespoke cocaine aIAT was administered to 46 volunteers in ecological settings, and paradoxical results (high number of false positives) were obtained from the comparison group. Quantitative data analyses excluded the presence of interfering factors, therefore a subsequent phase was embedded within the research design. The goal of the second research phase was in fact to explore if the testing technique did measure the presence of an autobiographical event in memory, or if it was a measurement of yet another cognitive construct [29]. The aIAT was re-administered (assessing heroin consumption this time) and life-story interviews were utilized to explore the participants’ autobiographical experience with cocaine and heroin. It was chosen to assess heroin being its use very rare within the populations chosen for this study [30]. The use of ethnographic methodology implying participant observation, as well as an analysis of the socio-demographic information regarding the sample, provided a general understanding of these individuals’ background, resulting in an increased reliability of autobiographical narrations [31].

### Interpretative process

**Figure 1:**
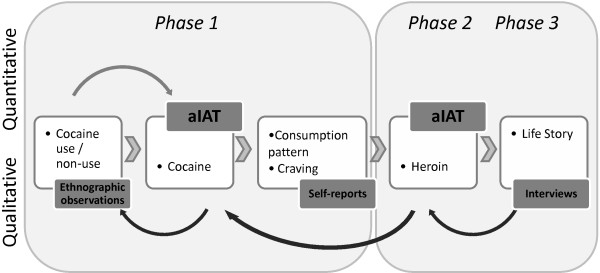
**The iterative and incremental research process.** Phases represent the temporal sequence of the study, whereas the boxed details describe the procedure followed by the interpretative process.

As shown in Figure 1, to integrate data derived from different measurement techniques, the study followed an embedded design which promoted a processual research plan [28]. The interpretative process integrated through subsequent but interdependent phases, a qualitative strand to a quantitative research design. In the first phase, information on cocaine craving and consumption patterns was analysed to exclude the hypothesis which attributed differences in performance to cocaine abuse [16]. Cocaine craving was assessed using a validated cocaine craving scale, whereas data regarding consumption trajectories were collected with open questionnaires and ethnographic note taking. The second phase compared quantitative analyses of performance scores, while the third phase implied the triangulation of qualitative data. Self-reports of drug experience and ethnographic observations were compared to test accuracy in order to explain the high number of false positives.

### Measurements

#### The brief cocaine and heroin aIAT

The test administered to the participants was custom designed following the aIAT instructions [8], using however a brief version of the IAT [26], which can be equally used to gain reliable results while dramatically reducing testing times and participant distress. The brief IAT has two blocks of trials with the same mappings as the standard IAT but with 1/3 the number of trials. Validation studies demonstrate how this version of the IAT has satisfactory validity, especially when the *self* is a focal category [32], as in the case of the items of an aIAT.

The aIAT, adapted for utilization on a portable computer with touch screen technology (an RM Slate with Windows 7 operating system), was created using Inquisit® software. The Brief IAT template was programmed for digital use by displaying a blue button on the right hand side for congruent matching and a green one on the left for incongruent pairing. The block’s pairing assignment, which described whether the task was to associate cocaine/heroin user or not a cocaine/heroin user with ‘True’ (congruent pairing), was presented on the upper part of the screen during the entire duration of the task.

The items, which were presented in the centre of the screen, followed Sartori and colleagues’ guidelines [8]. These were to utilise statements which are short and that represent mutually excluding events. Furthermore, validation studies suggested the detrimental effect of using negative statements as aIAT items [19]. We decided to investigate this condition in our study and randomly administered two different typologies of cocaine aIAT to participant groups: one which utilised negative statements to assess the condition of non-user (i.e. *I don’t use cocaine*) and one which used affirmative statements (i.e. *I keep off cocaine*) to assess the same condition.

During the third phase of the study which was embedded to verify the test’s construct validity, a heroin aIAT was created by replacing the word ‘cocaine’ with ‘heroin’ to the affirmative statement attribute category. As seen in Table 1, during the heroin aIAT administration, the negative statement version was not utilised for the reassessed sample. Four categories of autobiographical events (true; false; drug user; non-user) containing each four items refer to two different targets and two attributes: the generally true and false *target* categories refer to the individual’s setting, while the *attribute* categories refer to the individual’s status of cocaine/heroin user or non-user.

**Table 1:** Category labels and stimuli of the brief cocaine- and heroin-aIATs

**Items for Attribute A ‘True’:**		**Items for Attribute B ‘False’:**	
1	I’m in London	1	I’m climbing a mountain
2	I’m taking a test	2	I’m at the beach
3	I’m in front of a computer	3	I’m playing football
4	I’m reading the screen	4	I’m at a shop
Items for Target A ‘as if you were a cocaine user’:		Items for Target B (**negative** sentences) ‘as if you were not a cocaine user’:	
1	I’ve used cocaine recently	1	I don’t use cocaine
2	I use cocaine	2	I’ve never tried cocaine
3	I’ve snorted cocaine with friends	3	I don’t snort cocaine
4	I snort cocaine	4	I don’t use cocaine with friends
		Items for Target B (**affirmative** sentences) ‘as if you abstain from cocaine use’:	
		1	I am cocaine free
		2	I refuse to use cocaine
		3	I respect the law on cocaine use
		4	I keep off cocaine
Items for Target A ‘as if you were a heroin user’:		Items for Target B ‘as if you abstain from heroin use’:	
1	I’ve used heroin recently	1	I am heroin free
2	I use heroin	2	I refuse to use heroin
3	I’ve snorted heroin with friends	3	I respect the law on heroin use
4	I snort heroin	4	I keep off heroin

The first block (20 trials) of the test is fixed and presents only items from target categories. The remaining four blocks (two congruent and two incongruent) constitute the sorting task and are randomly assigned via the software program. The brief IAT template measures response times between congruent blocks (Attribute A with Target A; i.e. ‘True’ with ‘*as if you were a cocaine user’*) and incongruent ones (Attribute A with Target B; i.e. ‘True’ with ‘*as if you abstain from cocaine use*’). Through a validated algorithmic score incorporated in the software program [33] a D-score is obtained which summarizes the participant’s matching preference. *D* is calculated as the difference between mean latencies of two b-IAT blocks divided by the inclusive standard deviation of these two latencies [26]. In the present test setup, positive scores indicate preference associating the ‘A’ target and attribute categories (cocaine user is true) while negative scores indicate faster response times for incongruent blocks (not a cocaine user is true).

Phrasing attribute categories ‘as if you were a cocaine/heroin user’ and ‘as if you were not a cocaine/heroin user’ aimed to resolve a difficulty that many participants initially faced when performing incongruent pairing (in the case for example of the cocaine using group, matching ‘True’ with ‘not a cocaine user’), avoiding confusion between personal drug-using status and sorting task instructions. Further adaptations regarded the use of aIATs in Italian as the cocaine group was prevalently formed by mother tongue Italian individuals. Literature suggested language typology did not influence test performance [6,34]. Additional file 1: Table S1 presents the Italian version of the brief cocaine- and heroin-aIATs.

#### Craving and consumption trajectories

Managing craving, which is a desire of varying intensity to consume a substance, requires effort and consumes cognitive resources, thus it affects attention, concentration, memory retrieval and perception outside people’s conscious awareness [35]. Various research involving implicit measures and cocaine use investigating approach-avoidance, attentional processes and implicit associations suggests that cocaine abuse can influence cognitive processes and decision-making [36-39].

In view of the scoring differences found in original literature between drug abusing groups [8], possible correlations between consumption style and performance on the aIAT was also explored. After the autobiographical cognitive task, participants filled in a short questionnaire containing three open questions regarding the frequency of cocaine use: 1. *How many times have you approximately used cocaine during the last six months? 2. How many times, on average, have you used cocaine in the last month? 3. When was the last time you used cocaine?* Participants were assisted by the researcher when providing answers to these open questions, in order to collect sufficiently descriptive and interpretable data.

To explore the existence of any correlation between testing performance and cocaine craving, the Brief Cocaine Craving Questionnaire (CCQ-Brief) [40], a measure of the desire to use cocaine, was filled in by participants. The CCQ-Brief is a ten-item questionnaire with a 7-point Likert-type response scale which measures the agreement to statements referring to the urge to use cocaine. Examples of the items used in this questionnaire are: “*I want cocaine so bad I can almost taste it*”; “*I think I can resist using ‘coke’ now*” and “*I will use cocaine as soon as I get a chance*”. The CCQ-Brief was used as a general indicator of the participant’s urge to use cocaine at the time they completed the cocaine aIAT task, keeping its descriptive limitations in mind.

#### Life-story interviews

In order to collect in-depth data regarding the actual autobiographical experiences with drug using of the 23 participants of the second study, a life-story interview was conducted. The interview lasted approximately 15 minutes and was carried out after completing the aIAT sorting task. Participants were asked to describe their personal experience with the substances assessed (cocaine and heroin) and their general knowledge regarding these substances. Care was given in guiding the participant towards an autobiographical description of their relationship to the substances by using the Life-Story interview technique [41]. In particular, our interest was avoiding collecting only data relative to social representations and attitudes towards socially disapproved behaviours. Rather, the aims of the interview were to confirm self-reports referring to drug using status, and record any type of direct experience the subsample of participants had had with heroin and cocaine.

Note taking and Grounded Theory (GT) methodology were used for data collection and analysis. GT uses inductive-deductive interpretation which helps the researcher make sense of qualitative raw data through a circular process [25]. Considering sense-making as a social construction, quality and relevance of the data strongly depend on the active participation both of the researcher as of the population being researched. According to Atkinson [41], creating a co-participating relationship with interviewees is possible if value is given to the individual’s personal life experience. This way, it was possible to collect more reliable qualitative data less conditioned by participants’ resistance and suspicion. An open-minded, non-judgmental attitude was also vital and maintained during the whole data gathering process.

### Participants

The experimental group were 23 young adults (18–35 years old) living in Greater London (UK) who use cocaine for recreational reasons [42]. Recreational cocaine users usually present themselves as ‘socially integrated’, with an occupation and a secure socio-economic background [43]. In this population, individuals often engage in poly-drug use, and continuation of drug experimentation seems to prolong during their lifetimes [44]. Usually, these individuals are not ‘statistically visible’ not being in contact with healthcare institutions; they also maintain their behaviour anonymous to avoid legal consequences [43].

A main issue in drug research is limitation in the populations assessed in empirical research, where mainly recruitment in clinical settings is utilized, thus only populations of ‘pathological’ consumers are explored [45]. On the contrary, individuals in this study were recruited through ethnographic observations. These were carried out by a bilingual researcher who contacted a group of Italian speaking key individuals willing to co-participate in the research process. The status of ‘recreational’ cocaine user was self-attributed by the participant; nonetheless, special care was taken in contacting individuals who had been observed by the ethnographic researcher as utilizing the substance and maintaining the study sample within the same social network of users. Gender distribution (17 males; 6 females) resembled real life with males dominating the cocaine user group [30].

The comparison group of 23 non-cocaine and heroin users (15 females and 8 males) was recruited among postgraduate students. In line with recruitment of the cocaine user experimental group, the researcher contacted individuals as potential non-users via theoretical and snow-ball sampling [25]. Also, this sample was selected to match the age group of the cocaine users (cocaine group mean age = 26.91±3.50; comparison group mean age = 26.48±3.91).

The subsample of 10 cocaine users and 13 non-users who participated in the second phase of data collection was selected from the original study group. Individuals were assessed with a qualitative interview to explore possible correspondence with test results, and confirm self-declared and observed drug using status.

### Procedure

Forty-six individuals (25 males and 21 females) were asked if they had ever tried cocaine in their lifetime and if they wished to ‘co-participate’ through their collaboration and honesty to the research project. This approach was believed to be fundamental for the acquisition of reliable and truthful data from hidden populations [21]. The study received ethical approval from the Faculty Research Ethics Committee of the Faculty of Science, Engineering and Computing, Kingston University.

Once informed consent was obtained, the participant was accompanied to a quiet area and assisted in the comprehension and execution of the test and questionnaire. The sorting task for cocaine use detection was randomly administered according to statement typology (affirmative/negative statements) and using two conditions (cocaine user and comparison group). The CCQ-Brief and short questionnaire were administered after taking the brief cocaine aIAT.

The heroin brief aIAT was administered to 23 individuals (10 cocaine users and 13 nonusers) invited from the original participant groups and used to evaluate the test’s construct validity. No information was provided regarding differences in the substance assessed by the test until actual re-administration. Providing as little *a priori* information as possible regarding true aims and objectives of an IAT increases efficacy of this indirect measurement [6]. Once testing was completed, the researcher described the actual aims and objectives of the study, and carried out the Life-story interview [41] which lasted 15 minutes approximately.

### Data analysis

#### Quantitative data

Results of the randomly administered brief cocaine/heroin aIATs were analysed using two dependent measures (mean latency in the double-categorization blocks and D indices). Statistical analyses of mean D-scores were used to examine within and between group correlations using SPSS 19.0 software and r-to-z conversion for direct comparison of the correlation coefficients from the two independent groups. Correlation coefficients (Pearson’s r/ Spearman’s r and χ^2^ with Fisher’s Exact Test significance) were used to detect associations, while accuracy was measured through ROC analysis. Significance level was set at p < 0.05, nondirectional for all statistical analyses. Group differences were tested using Student t-test and mixed model ANOVA where drug-use group and test typology were treated as fixed, whereas language and gender as random effects. Effect sizes are presented as Cohen’s d and partial η^2^.

#### Qualitative data

Raw data derived from the open questionnaire were categorized *post hoc*. Consumption style and past use categories for the cocaine using group were defined by integrating reported use and longitudinal observations collected ethnographically*.* Qualitative data from interviews were integrated at a final stage and compared with test performance, categorizing scoring as accurate or inaccurate. Grounded theory methodology was used to analyse and order qualitative raw data through an inductive-deductive interpretative process [25], providing a rich context in which the aIAT results could be correctly interpreted.

## Results

### Language and test typology differences

Although the aIATs used in this study were completed by a mixed group of Italian and English participants, each using a version in their respective native languages, this variation did not result in statistically significant differences. Means, dispersions and test statistics are presented in Additional file 2: Table S2: Language, gender and typology differences. As would be expected from previous attempts [8,19,34], these results provided reassurance that language variations were not having an effect on the aIAT outcome measures.

The remaining question was whether statement typology affects the aIAT outcome. According to Sartori and colleagues [19], using affirmative statements to discriminate autobiographical events increases the accuracy of the lie-detector technique. In the study by Verschuere et al. [34], where the aIAT showed lower accuracy (64%), the classification of ‘innocent’ participants as guilty was attributed to the use of negative sentences for the innocent statement category [19]. According to the authors, this higher salience of negative statements would be explained through the figure-ground model of Rothermund and Wentura [46]. In brief, this model challenges the underlying assumption that IAT results are reflections of the subconsciously held associations between target concepts and competing bipolar attributes; and posits that instead, these are dependent on salience asymmetries.

Our results did not confirm this indication concerning the test’s functioning. Contrarily to the accuracy rates indicated [19], we did not find any differences between the negative statement and affirmative statement cocaine aIATs. The total accurate test results (Table 2) suggested that sentence typology had no influence on test accuracy (for further details and statistical test results, see Additional file 2: Table S2: Language, gender and typology differences). Following the figure-ground model [46], higher efficacy of the affirmative cocaine aIAT should have been more evident within the comparison group (being more susceptible to false positive categorization) but again, no significant differences in accuracy were found between test types (χ^2^ = 0.087; p > 0.999). It was therefore necessary to hypothesize that other order of factors existed which influenced the aIAT’s efficacy.

**Table 2:** Cross-tabulation of classification and statement typology

	**Accurately classified**	**Inaccurately classified**	**Total number of participants (100%)**
**Negative statement**	11 (57.8%)	8 (42.1%)	19
**Affirmative statement**	17 (62.9%)	10 (37%)	27
**Overall**	28 (60.9%)	18 (39.1%)	46

### Accuracy of the cocaine aIAT

Having found no significant differences for language, statement typology or gender, cocaine use remained our primary independent variable and qualitative measures were integrated within the experimental design. The overall accuracy of the aIAT, including both known cocaine users and non-users was 61%. This is lower than previously reported by Sartori et al. [8,15,19], but in line with the 64% obtained by Verschuere et al. [34]. Ethnographic studies are expected to yield lower accuracy compared to controlled laboratory studies owing to the increased variability.

**Figure 2:**
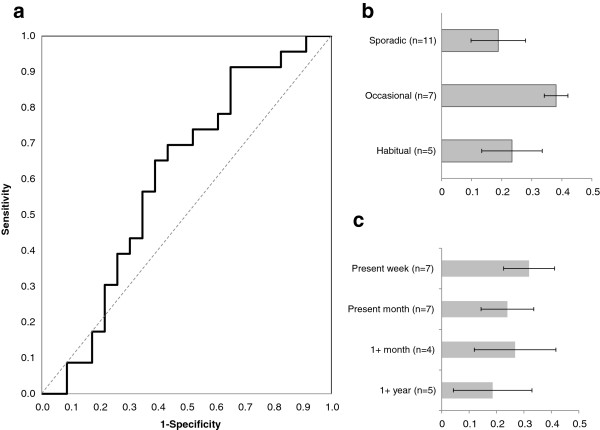
**Cocaine aIAT: Accuracy and differences in D-scores by consumption style and last use.** (**a**) Area under the Curve. (**b**) Bar chart depicting mean D-scores categorized by consumption style for the cocaine using groups of sporadic users (D = 0.22), occasional users (D = 0.30 and habitual users (D = 0.23). (**c**) Comparison of D-scores between those who used cocaine within a week (D = 0.31), during the last month (D = 0.18), within a year (D = 0.26) and more than a year from test administration (D = 0.28). Whiskers represent SEM.

However, when administering the aIAT to the comparison group of 23 non users, the test’s invalidity emerged immediately. Means accounting for D-scores revealed the test’s lack of specificity. Average D-scores for the recreational cocaine users (n=23) was 0.257± .246, while for the comparison group (n=23) mean D-score reached 0.134±.367, showing no discrimination between cocaine users and abstinent participants [t(44) = 1.339, p = 0.187, Cohen’s d = 0.395]. The aIAT incorrectly classified 14 (60.8%) non-users as cocaine users. Considering that 18 (78.3%) cocaine users were accurately detected, the aIAT appears to show reasonably good sensitivity, but very mediocre specificity. The AUC value for our empirical dataset (Figure 2a) using cocaine-aIAT D-scores was 0.605 (95% CI 0.437, 0.773) for the cocaine user group and conversely, only 0.395 (95%CI 0.231, 0.559) for the nonusers. The AUC value of 60.5% was well below the accuracy scoring previously reported [8] and as indicated by the p-value (0.223), classification of cocaine users and non-users was not statistically better than classifying by pure guessing. The ROC curve parameters are provided in Additional file 3: Table S3: ROC analysis.

### The effect of situational influence: craving and consumption

When describing craving and consumption measures, it was hypothesized that attentional bias and cocaine implicit associations [16,36,37,47] might have had an influencing role in determining differences in D-scores within Sartori’s drug detection study [8]. We did not find any connection between cocaine consumption and craving rates, and test accuracy (D-scores). As predicted, cocaine craving scores correlated positively with recent cocaine use (r_S_(23) = 0.475; p = 0.022) and consumption style (r_S_(23) = 0.576, p = 0.004) but not with the aIAT results (r_S_(23) = 0.115, p = 0.601). This absence suggests that the retrieved association is person- rather than situation-based.

Figure 2b shows that no differences in mean D-scores were found between sporadic cocaine users (defined as having used cocaine less than 5 times in 6 months), occasional users (having used cocaine once a month) and the every weekend habitual users (F(2,14) = 0.765, p = 0.484, η^2^ = 0.099). Figure 2c compares groups of cocaine users according to their last reported use of cocaine. No statistically significant difference was found in D-scores (F(3,14) = 0.283, p = 0.837, η^2^ = 0.057) and there was no interaction effect between consumption style and recent reported use on the test’s D-scores (F(3,14) = 0.229, p = 0.875).

In summary, although the cocaine aIAT was modelled closely to the original aIAT [8] and Sriram & Greenwald’s brief IAT [26], it was not able to reliably discriminate between cocaine users and non-users. The lack of relationships between D-scores and consumption measures led us to further investigate aIAT performance, triangulating qualitative data to further explore the unexpected rate of false positives. In Study 2, we integrated a re-test with an aIAT for heroin use (a drug that was clearly absent from both experimental groups) and qualitative data from life-story interviews.

### What does the aIAT test measure? – insights from the heroin aIAT

The second phase of data collection regarded a re-test using a heroin aIAT administered to a subsample of the initial study group (n=10 cocaine users, n=13 comparison group); all declared to have never used heroin. Interestingly, the heroin aIAT was able to identify the cocaine using group as heroin abstainers but not the comparison group: average heroin D-score for cocaine users was −0.112±0.284 and 0.01±0.444 for the controls. Although the mean D-scores are relatively low, these ranged between −0.449 to 0.254 in cocaine users and between −0.576 to 0.744 in controls.

**Figure 3:**
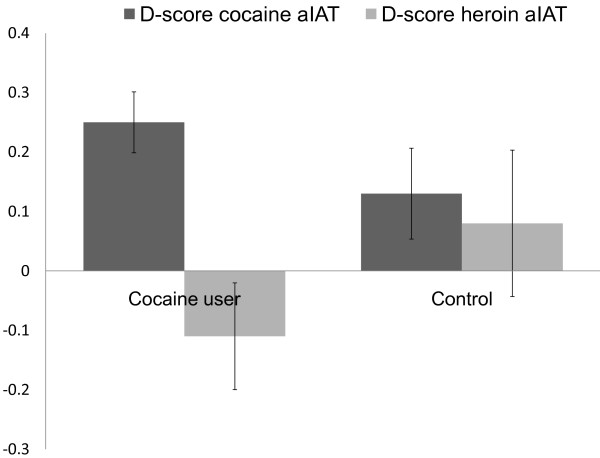
**Mean D-scores obtained by cocaine users and non-user comparison sample on brief cocaine- and heroin-aIATs.***Legend:* Negative scores represent preference matching Abstainer with True, while positive scores indicate faster associations for the User and True category.

In Figure 3, outcome trends evidence that cocaine users were recognized as heroin abstainers whereas non-drug users showed again, a profile that would be expected from drug users. The difference, although graphically more noticeable between cocaine users’ and non-users’ D-scores, was not statistically significant [t(21) = −0.756, p = 0.458, Cohen’s d = 0.274].

Overall D-scores obtained in the cocaine and heroin aIATs showed a statistically significant difference in the cocaine user group [D_C_ = 0.248±0.297 vs. D_H_ = −0.112±0.284; t(9) = 2.855, p = 0.019] but not in the comparison group [D_C_ = 0.158±0.413 vs. D_H_ = −0.010±0.444; t(12) = 1.851, p = 0.089]. Based on these results we assumed that the aIAT might tap into some general associations on social drugs or it may not always activate semantic or autobiographical memory, consenting prevarication of other mechanisms which led to the observed ‘false’ positive outcomes.

**Figure 4:**
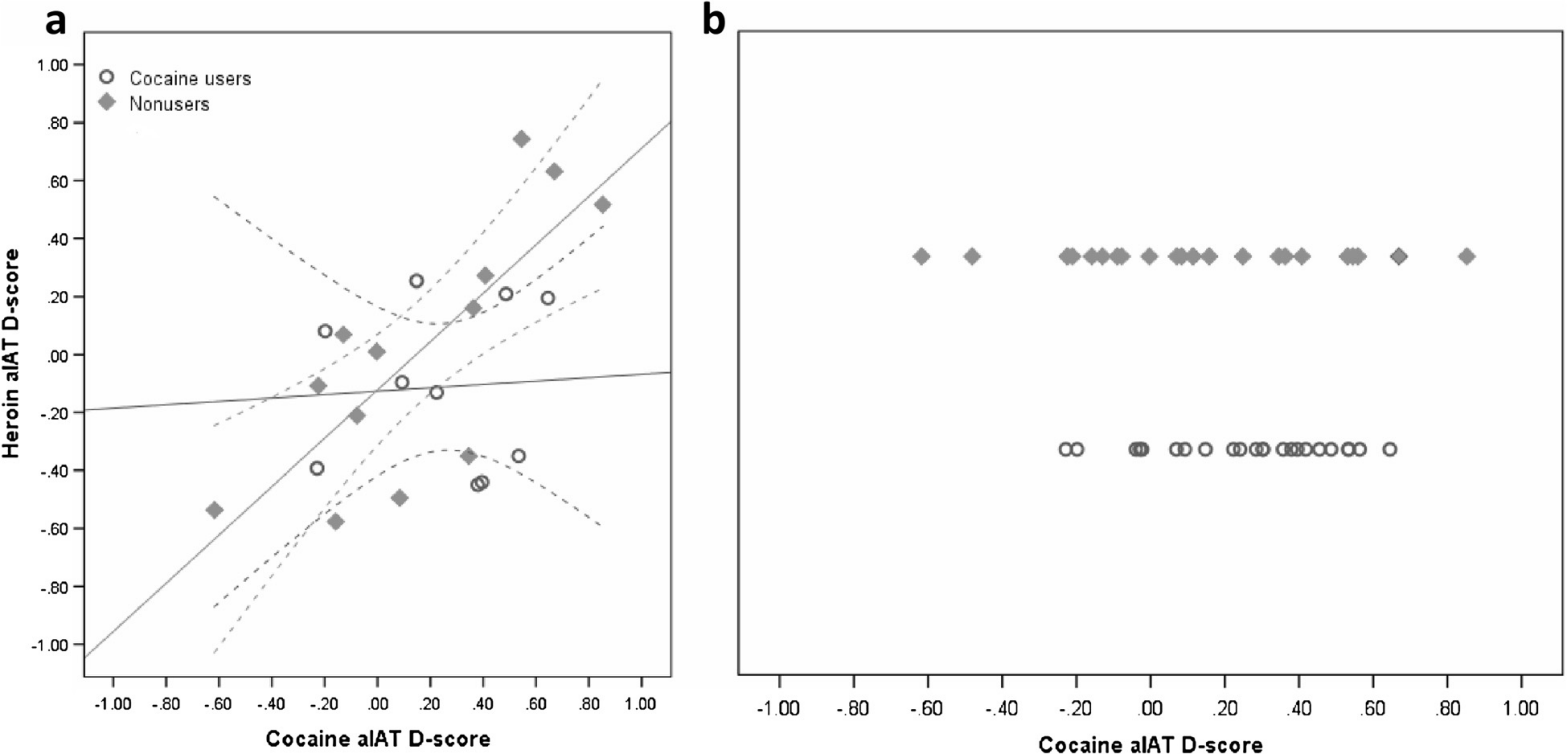
**Individual cocaine and heroin D-scores.** (**a**) Relationship between cocaine and heroin D-scores; line represents the simple linear regression model fitted to the group data independently, with 95%CI of the mean. (**b**) Dispersion of cocaine D-scores in the cocaine user and non-user comparison groups.

Coherently to this principle, we found an unexpectedly strong correlation between the cocaine and heroin brief aIATs in the non-user group (r(13) = 0.776, p = 0.002) coupled with the absence of such relationship (r(10) = 0.061, p = 0.866) in the cocaine user group. Figure 4a shows the relationship between cocaine and heroin D-scores. Comparing the two independent correlation coefficients directly using a z transformation, the correlation was significantly stronger for the comparison group than for the cocaine users (z = 3.081, p = .0021). It would seem that being more ‘drug experienced’, the recreational cocaine using group performs more accurately on the associative task, while comparison groups’ performance seems to be more linked to an IAT effect, leading to constancy in their scoring. The dispersion of individual cocaine D-scores among non-users (Figure 4b) shows a wider spread and significant overlap with the D-scores obtained from cocaine users, evidencing the lack of specificity of the aIAT in this setting.

If in fact, intra-test performance is observed between groups (D-scores in the first two test blocks compared to the third and fourth of the sorting task) strong differences are observed as well. While mean D-scores increase significantly between block groups in the comparison group (1^st^ block mean D = 0.057±0.414, 2^nd^ block D = 0.217±0.425) showing positive correlation (r(23) = 0.531; p = 0.009), this is not observed in the cocaine group (1^st^ block mean D = 0.241±0.407; 2^nd^ = 0.273±0.333; r(23) = −0.127; p = 0.563). The same intra-test performance trend is observed in the heroin aIAT. Similarly to the previous test, comparison group’s preference for the congruent block increases during the task, while cocaine users show a slight improvement when pairing the incongruent block (*not a heroin user is true)*. It seems that the high rate of false positives in the comparison group could be potentially created at the moment by an ‘IAT effect’, or association transfer [29], as will be discussed in the following paragraphs. Mean response times in congruent and incongruent blocks for both cocaine and heroin aIAT are presented in Additional file 4: Table S4: Mean response times of the congruent and incongruent blocks.

### Life-story interviews

The analysis of qualitative data from life-story interviews compared autobiographical events to aIAT performance. As already underlined, the interview aimed to collect information regarding ‘drug experience’ rather than solely exploring drug attitudes. This qualitative string integration had a determining role in our understanding of the paradoxical findings. From the triangulation of autobiographical narrations and aIAT results parallels emerged, especially when extreme cases were analysed: participants least suspected of having experience with illicit substances (due to religious views and cultural background), obtained the highest positive scores on both tests. Comparison group participants who did obtain accurate scores usually described a negative direct experience with cocaine/heroin users. For example, a participant referred of her distrust towards illicit substance use because of a sister who suffered from drug dependency. Another participant who scored accurately on the aIAT was currently living with drug users and described these substances as “*leading to death*”.

These comparison group participants who were falsely categorized as drug users on both tests disapproved illicit substance use but had “*never encountered these drugs before, except on films*”. These participants showed scarce knowledge of the implied substances. Similarly, cocaine group participants who were not categorized accurately (obtaining either negative scores on the cocaine aIAT or positive scores on the heroin aIAT) were almost entirely self-reported sporadic users, whose last use of cocaine went back to more than a year from taking the test.

Regarding attitudes and social representations emerging from the narrations, the sample presents a predictable scenario: both recreational cocaine users and the majority of the comparison group considered cocaine less dangerous than heroin. The latter was usually described as lethal and no one expressed curiosity towards experimenting with it, unlike cocaine.

If D-score averages of cocaine and heroin aIATs (0.19 and −0.08 respectively) are observed, it may be inferred that attitudes play a role in determining inaccurate scores. But a more accurate analysis which involves the participants’ direct experience evidences this aspect as more predictive of the test’s specific accuracy. Therefore, the score obtained on the aIAT may be primarily influenced by the individual’s capacity of recalling a direct experience or semantic Self-concept referring to cocaine or heroin use, rather than by the actual presence of autobiographical memory related to cocaine/heroin use. A general discussion regarding the interpretation of the test’s functioning is presented in the following sections.

## Discussion

When administering the aIAT to the comparison group of 23 non-users, the test’s invalidity emerged immediately. Paradoxically, higher scores were obtained by individuals who, owing to cultural background or religious views, were the least suspected of having tried cocaine, or of having found themselves within a context where this substance was used. Notably, these person-specific considerations could not have been made if qualitative measures were not integrated within the experimental design. In fact, using an investigative process instead of an inquisitive approach was fundamental in understanding final results, especially when considering the ecological setting of the study [48].

The parallels between the individual’s autobiographical narrations and obtained D-scores led us to conclude that the aIAT might tap into some general associations people have referring to the target behaviour (i.e. drug use) which does not always correspond to autobiographical memory. The activation of other mechanisms might have led to the observed ‘false’ positive outcomes. In the case of our study, it appears that the associative task was more capable of tapping into the recreational cocaine users’ autobiographical memory when these were ‘drug experienced’, while comparison groups’ performance seemed to be more linked to an IAT effect (this can be inferred by the correlations found between tests and trial blocks).

The fact that the aIAT was not effective in accurately detecting drug use in the present study does not preclude the test being valid and reliable in other specific circumstances. In the case of our study, the test demonstrates reliable sensitivity but a concerning lack of specificity. This determines serious complications in the application of the aIAT technique within forensic settings, where the issue to address is normally the innocence or responsibility of an individual.

Sartori and colleagues recently identified a ‘neutral window’ for D-scores between −0.2 and 0.2 where accuracy falls below 80% [9]. In the cocaine user group of our study, 6/23 for cocaine aIAT and 3/10 for heroin aIAT fell into this window, whereas the ratios were 10/23 and 4/13 for non-user controls, respectively. Even after excluding respondents with D-scores between −0.2 and 0.2, the accuracy of the aIAT classification actually deteriorates (AUC = 0.511, p = 0.917). Although having obtained very similar rates for the heroin aIAT ‘neutral window’ in both groups, the aIAT’s efficacy was dramatically different in the two groups, and this caveat alone cannot explain the high rate of inaccuracy found in our study. Notably, the ratio for −0.2 ≤ cocaine D ≥ 0.2 was significantly higher in the comparison group compared to the users. This supports our proposed notion that in the absence of a relevant autobiographical event, the final score could be potentially created at the moment by association transfer, affecting reaction times and consequently D-scores.

This study also challenges Sartori’s attempt to explain aIAT functioning through Rothermund and Wentura’s figure-ground model [19]. Although sample size differences between the aforementioned and the present study limit our degree of confidence, salience asymmetry seems not to influence test accuracy, so it is improbable that this mechanism is involved in the test's overall functioning. Association theory remains the most commonly accepted explanation for IAT functioning and Sartori’s lie-detector is a variant of this technique [49]. Considering our results, it is possible that outcomes might have been undermined by the degree of involvement of the participants and/or by task instructions (i.e. how much does imagining the statement influence preference in matching?). As evidenced by Hu and Rosenfeld [12], propositional thinking (higher order conscious reasoning) can create new associations which influence associative processing.

If the associative assumption regarding IAT functioning is not completely dismissed, the Relational Frame [29] and the Social Knowledge Structure theories [50] can provide a plausible explanation for the comparison group’s improvement and constancy when pairing the congruent block. Assuming that the concepts elicited by the statements (“*I*” and “*using/not using cocaine*”) do not completely relate to semantic memory cognitively available, during the associative task preference in pairing first-person statements referring to drug use with ‘true’ would be translated into pairing a positive Self-concept (due to the absence of a concept available referring to the drug) with a positive attribute (True), by the principle of a conceptual entailment [29].

In the structuring of social knowledge, the ‘*Self*’ holds a central role and is assumed to be strongly associated with positive valence concepts. This would result in a preference in pairing the Self with positive focus categories during IAT [50]. It can be assumed that in the absence of autobiographical memory or of any kind of semantic knowledge relative to substance use, our aIAT may measure a response deriving from a positive Self-concept (*I am using)* with a positively charged attribute (True). The importance of the Self-concept in associative knowledge could perhaps explain aIAT phenomenology more accurately.

In Sartori and colleagues’ studies [8,15,19], *forma mentis* and methodological choices might have contributed to the apparent higher accuracy of this testing technique. It is generally accepted that implicit measures are determined by automatic responses deriving from attitudes whose origin are unknown, and coexist with more deliberative processes [18]. When considering autobiographical events, memory of these may coexist with several other decisional and affective processes [51]. Substance abuse may also influence cognitive processes and social knowledge can lead to strong attitudes which often undermine objectivity.

Triangulating quantitative results with qualitative information provided a rich context in which assumptions supporting the aIAT concept could be challenged. The use of a mixed methodological approach provided an alternative theoretical framework for the interpretation of quantitative data and thus was valuable in understanding what the aIAT measures in real life settings.

As it is often difficult to gain reliable data concerning socially disapproved conducts and validate participants’ drug using status through biological markers (especially when considering an individuals’ entire lifetime), the use of ethnographic methodology for sample recruitment was fundamental to remedy limitations. This approach in fact, ensures that a privileged relationship is created with participants and value is given to subjective experience [21]. Finally, the integration of a qualitative strand in the research design provided an alternative theoretical framework for the interpretation of confounding results [52].

## Conclusions

The brief version of the aIAT was created and adapted for touch screen application to gain reliable data while dramatically reducing testing times and participant distress. The brief cocaine- and heroin-aIATs successfully identified the recreational cocaine using group’s status with accuracy comparable to previous studies, but failed when identifying more than half of the comparison group as heroin and cocaine users. The lack of relationship between cocaine use measures and aIAT performance, as well as the correlations found in the comparison group’s scores indicated that the aIAT is not situation- but person-dependent.

Furthermore, the key finding of this study was the aIAT’s concerning inaccuracy in correctly identifying ‘not guilty’ individuals in ethnographic settings. Further investigation is needed to fully explore the test’s functioning. Nonetheless, based on our results from mixed methodology, it is conceivable that if the autobiographical event is not available to the tested individual, aIAT outcomes may be severely influenced by propositional thinking or task instructions, thus results may not be exclusively dependent on automatic associations [18,29]. Moreover, implicit attitudes and self-esteem [50] may also play a crucial role and their influence should be investigated. It is possible, for example, that association transfers between semantic concepts may lead to false positive outcomes. Before anything else, one vital issue to address is the identification of a correct operational definition of ‘*autobiographical event*’.

By applying the falsifiability criterion [53], this study successfully underpinned theoretical and conceptual pre-assumptions which probably led to an overestimation of the aIAT’s accuracy and reliability. Considering the vastness of literature which confirms the test’s validity and the reassuring sensitivity demonstrated in the present study, confounding results should not discourage the application of this instrument in diagnostic settings. Owing to the attractiveness of having a fast, portable, and economical tool to identify ‘guilty’ in a large unknown population, it is expected that the response-time based ‘lie-detectors’ will continue to attract attention of researchers and practitioners alike. More research into the aIAT’s functioning is strongly advised before further forensic use of the instrument, to ensure that vicarious experiences and other mental associations do not confound results.

## Competing interests

The authors declare that they have no competing interests.

## Authors’ contributions

AP designed the study and wrote the protocol with EJV. EJV performed the literature search, developed the brief aIAT, adapted the aIAT tests for touchscreen application, collected the data, contributed to data analysis and drafted the first version of the manuscript. AP conducted the statistical analysis and contributed to writing the manuscript. Both authors contributed to and have approved the final manuscript.

## Supplementary Material

Additional file 1: Table S1 Italian version of the brief cocaine- and heroin-aIATs. Italian translation of the category labels and stimuli of the brief cocaine- and heroin-aIATs. Click here for file

Additional file 2: Table S2 Language, gender and typology differences. Detailed statistical analysis for language, gender and typology differences. Click here for file

Additional file 3: Table S3 ROC Analysis. Curve parameters of the ROC analysis. Click here for file

Additional file 4: Table S4 Mean latencies of the congruent and incongruent blocks. Mean latencies (in ms) ± SD of the congruent and incongruent blocks obtained in the brief cocaine- and heroin-aIATs. Click here for file
