# Supplementary material for: Detecting cocaine use? The autobiographical implicit association test (aIAT) produces false positives in a real-world setting
Source: Subst Abuse Treat Prev Policy. 2013 Jun 14;8:22. doi: 10.1186/1747-597X-8-22 (PMC3685584; doi:10.1186/1747-597X-8-22)
Supplement: Additional file 1: Table S1 — Italian version of the brief cocaine- and heroin-aIATs. Italian translation of the category labels and stimuli of the brief cocaine- and heroin-aIATs. [file 1747-597X-8-22-S1.pdf]

Italian translation of the category labels and stimuli of the brief cocaine- and heroin-aIATs

Items for Attribute A 'Vere':		Items for Attribute B 'False':	
1	Mi trovo a Londra	1	Sto scalando una montagna
2	Sto facendo un test	2	Sono al mare
3	Sono davanti al PC	3	Gioco a calcio
4	Guardo uno schermo	4	Mi trovo in un negozio
Items for Target A 'come se fossi un consumatore di cocaina':		Items for Target B (*negative* sentences) 'come se non fossi un consumatore di cocaina':	
1	Ho usato cocaina recentemente	1	Io non uso cocaina
2	Consumo cocaina	2	Non ho mai provato cocaina
3	Ho sniffato cocaina con i miei amici	3	Non sniffo cocaina
4	I uso cocaina	4	Non uso cocaina con i miei amici
		Items for Target B (*affirmative* sentences) 'come se ti astenessi da usare cocaina':	
		1	Sono libero dall'uso di cocaina
		2	Mi tengo lontano dalla cocaina
		3	Rispetto la legge sulla cocaina
		4	Evito la cocaina
Items for Target A 'come se fossi un consumatore di eroina':		Items for Target B 'Come se ti astenessi da usare eroina':	
1	Ho usato eroina recentemente	1	Sono libero dall'uso di eroina
2	Consumo eroina	2	Mi tengo lontano dall'eroina

3	Ho sniffato eroina con i miei amici	3	Rispetto la legge sull’eroina
4	I uso eroina	4	Evito l’eroina
