# Supplementary material for: Detecting cocaine use? The autobiographical implicit association test (aIAT) produces false positives in a real-world setting
Source: Subst Abuse Treat Prev Policy. 2013 Jun 14;8:22. doi: 10.1186/1747-597X-8-22 (PMC3685584; doi:10.1186/1747-597X-8-22)
Supplement: Additional file 2: Table S2 — Language, gender and typology differences. Detailed statistical analysis for language, gender and typology differences. [file 1747-597X-8-22-S2.pdf]

Detailed statistical analysis for language, gender and typology differences

Table S.1.

Means ± SD for language, test type and gender differences

	Typology	Random factors	N	Study 1: Brief cocaine-aIAT		N	Study 2: Brief heroin-aIAT	
				Latency (ms)	D score		Latency (ms)	D score
Cocaine user	Negative	Italian	4	2294.32 ± 217.66	0.373 ± 0.063		-	-
		English	5	2108.07 ± 753.85	0.133 ± 0.278		-	-
	Affirmative	Italian	12	2817.94 ± 2680.06	0.239 ± 0.270		-	-
		English	2	1636.64 ± 525.11	0.441 ± 0.064		-	-
	Negative	Male	6	2160.58 ± 323.34	0.363 ± 0.132		-	-
		Female	3	2251.38 ± 988.21	-0.005 ± 0.213		-	-
	Affirmative	Male	11	2787.31 ± 2832.00	0.198 ± 0.244		-	-
		Female	3	2142.73 ± 498.22	0.525 ± 0.125		-	-
	Total	Negative	9	2190.85 ± 558.16	0.240 ± 0.237		-	-
		Affirmative	14	2649.18 ± 2506.57	0.268 ± 0.260		-	-
		Italian	16	2687.03 ± 2309.03	0.273 ± 0.241	4	1526.42 ± 190.98	-0.186 ± 0.297
		English	7	1973.38 ± 691.18	0.221 ± 0.273	6	1556.73 ± 305.94	-0.063 ± 0.292
		Male	17	2566.11 ± 2267.29	0.256 ± 0.222	6	1501.52 ± 239.42	-0.138 ± 0.311
		Female	6	2197.06 ± 702.46	0.25994±0.329261	4	1609.25 ± 297.29	-0.074 ± 0.279
	Grand total		23	2469.83 ± 1969.32	0.25701±0.245947	10	1544.61 ± 253.77	-0.112 ± 0.284

	Typology	Random factors	N	Study 1: Brief cocaine-aIAT		N	Study 2: Brief heroin-aIAT	
				Latency (ms)	D score		Latency (ms)	D score
Non-user	Negative	Italian	1	2011.09	-0.003		-	-
		English	9	2050.06 ± 661.10	0.142 ± 0.288		-	-
	Affirmative	Italian	3	1779.08 ± 594.68	-0.009 ± 0.646		-	-
		English	10	1392.18 ± 325.15	0.182 ± 0.388		-	-
	Negative	Male	1	2011.09	-0.003		-	-
		Female	9	2050.06 ± 661.10	0.142 ± 0.289		-	-
	Affirmative	Male	7	1420.47 ± 174.19	-0.077 ± 0.392		-	-
		Female	6	1552.63 ± 594.26	0.389 ± 0.361		-	-
	Total	Negative	10	2046.17 ± 623.41	0.128 ± 0.276		-	-
		Affirmative	13	1481.46 ± 408.68	0.138 ± 0.435		-	-
		Italian	4	1837.08 ± 499.22	-0.007 ± 0.527	4	1490.77 ± 244.82	-0.026 ± 0.493
		English	19	1703.81 ± 600.83	0.164 ± 0.336	9	1512.69 ± 500.95	0.026 ± 0.451
		Male	8	1494.29 ± 263.84	-0.067 ± 0.364	4	1417.33 ± 291.78	0.072 ± 0.524
		Female	15	1851.09 ± 662.94	0.241 ± 0.331	9	1545.33 ± 485.80	-0.018 ± 0.436
	Grand total		23	1726.99 ± 576.21	0.134 ± 0.367	13	1505.94 ± 427.08	0.010 ± 0.444

Table S.2.

Test statistics (F, p) for language, test type and gender differences

Models[a]	Study 1: Brief cocaine-aIAT latency (ms)				Study 1: Brief cocaine-aIAT D score			
	F	p	Partial η^2	Main effect η^2	F	p	Partial η^2	Main effect η^2
Model 1								
Typology	0.069	0.836	0.065	0.002	0.013	0.927	0.013	< 0.001
Language	1.770	0.410	0.639[a]		0.398	0.642	0.285[a]	
Typology * Language	1.135	0.293	0.026		0.869	0.356	0.020	
Model 2								
Typology	0.252	0.704	0.201	0.001	0.040	0.874	0.038	0.008
Gender	1.360	0.451	0.576		0.064	0.843	0.060	
Typology * Gender	0.240	0.627	0.006		8.994	0.005	0.176	
Language * Gender	0.800	0.376	0.019		1.014	0.320	0.024	
Status * Language * Gender[b]	0.170	0.682	0.004		0.354	0.555	0.009	

[a] Italian version is more verbose compared to the English, which was expected to have effect on latency but not the D scores.
[b] Due to the small sample size in some cells, three way interaction effect with typology cannot be tested. Owing to random sampling variation, cocaine users were dominantly Italian speaking males (14/23) whereas controls were dominantly English speaking females (13/23); with strength of language and gender associations for cocaine users and controls, respectively as $\chi^2 = 5.033$, Fisher's exact p = 0.045 and $\chi^2 = 0.494$, Fisher's exact p = 0.589.
