# Supplementary material for: Detecting cocaine use? The autobiographical implicit association test (aIAT) produces false positives in a real-world setting
Source: Subst Abuse Treat Prev Policy. 2013 Jun 14;8:22. doi: 10.1186/1747-597X-8-22 (PMC3685584; doi:10.1186/1747-597X-8-22)
Supplement: Additional file 3: Table S3 — ROC Analysis. Curve parameters of the ROC analysis. [file 1747-597X-8-22-S3.pdf]

Curve parameters of the ROC analysis

Probabilities of membership in the cocaine user group	Positive if cocaine aIAT D ≥	Sensitivity	1 - Specificity
.0000000	-1.61716	1.000	1.000
.2809582	-0.54906	1.000	.957
.3336634	-0.35496	1.000	.913
.3696441	-0.22674	.957	.913
.3723334	-0.21763	.957	.870
.3762443	-0.20442	.957	.826
.3840184	-0.17838	.913	.826
.3942584	-0.14429	.913	.783
.4046764	-0.10996	.913	.739
.4125723	-0.08408	.913	.696
.4203608	-0.05877	.913	.652
.4279598	-0.03413	.870	.652
.4304457	-0.02610	.826	.652
.4344294	-0.01328	.783	.652
.4489143	0.03310	.783	.609
.4604940	0.07012	.739	.609
.4627041	0.07715	.739	.565
.4661165	0.08800	.739	.522
.4709238	0.10326	.696	.522
.4756539	0.11827	.696	.478
.4810067	0.13523	.696	.435
.4865521	0.15280	.652	.435
.4984829	0.19054	.652	.391
.5117205	0.23243	.609	.391
.5157004	0.24502	.565	.391
.5223693	0.26616	.565	.348
.5308779	0.29314	.522	.348
.5338631	0.30262	.478	.348
.5405346	0.32387	.435	.348
.5490429	0.35098	.435	.304
.5517494	0.35962	.391	.304
.5552606	0.37086	.391	.261
.5604352	0.38745	.348	.261
.5648233	0.40155	.304	.261
.5683540	0.41292	.304	.217
.5756629	0.43656	.261	.217
.5861188	0.47052	.217	.217
.5977264	0.50856	.174	.217
.6048666	0.53205	.174	.174

.6052715	0.53339	.130	.174
.6070654	0.53934	.087	.174
.6108586	0.55194	.087	.130
.6135871	0.56102	.087	.087
.6263498	0.60413	.043	.087
.6419349	0.65701	.000	.087
.6709382	0.76054	.000	.043
1.0000000	1.85208	.000	.000
