# Supplementary material for: Detecting cocaine use? The autobiographical implicit association test (aIAT) produces false positives in a real-world setting
Source: Subst Abuse Treat Prev Policy. 2013 Jun 14;8:22. doi: 10.1186/1747-597X-8-22 (PMC3685584; doi:10.1186/1747-597X-8-22)
Supplement: Additional file 4: Table S4 — Mean latencies of the congruent and incongruent blocks. Mean latencies (in ms) ± SD of the congruent and incongruent blocks obtained in the brief cocaine- and heroin-aIATs. [file 1747-597X-8-22-S4.pdf]

Mean latencies (in ms) ± SD of the congruent and incongruent blocks obtained in the brief cocaine- and heroin-aIATs

		Cocaine aIAT	Heroin aIAT
**Cocaine users**	**Congruent block**	2104.846 ± 495.5039	1527.0195 ± 283.1881
	**Incongruent block**	2002.462 ± 524.9322	1456.1135 ± 201.4940
	**Latency**	2469.833 ± 1969.3219	1544.5962 ± 253.7711
**Non-users**	**Congruent block**	1689.505 ± 594.5628	1514.0174 ± 481.9225
	**Incongruent block**	1720.345 ± 598.6522	1485.1892 ± 375.965
	**Latency**	1726.988 ± 576.21	1505.939 ± 427.0726
